# Clinical manifestations of dengue in relation to dengue serotype and genotype in Malaysia: A retrospective observational study

**DOI:** 10.1371/journal.pntd.0006817

**Published:** 2018-09-18

**Authors:** Jeyanthi Suppiah, Siew-Mooi Ching, Syafinaz Amin-Nordin, Lailatul-Akmar Mat-Nor, Naematul-Ain Ahmad-Najimudin, Gary Kim-Kuan Low, Manisya-Zauri Abdul-Wahid, Ravindran Thayan, Hui-Yee Chee

**Affiliations:** 1 Department of Medical Microbiology and Parasitology, Faculty of Medicine and Health Sciences, Universiti Putra Malaysia, Selangor, Malaysia; 2 Virology Unit, Infectious Disease Research Centre, Institute for Medical Research, Kuala Lumpur, Malaysia; 3 Department of Family Medicine, Faculty of Medicine and Health Sciences, Universiti Putra Malaysia, Selangor, Malaysia; 4 Malaysian Research Institute on Ageing, Faculty of Medicine and Health Sciences, Universiti Putra Malaysia, Serdang, Malaysia; 5 Microbiology Unit, Department of Pathology, Hospital Serdang, Selangor, Malaysia; 6 Department of Population Medicine, Faculty of Medicine and Health Sciences, Universiti Tunku Abdul Rahman, Selangor, Malaysia; Mahidol Univ, Fac Trop Med, THAILAND

## Abstract

**Background:**

Malaysia experienced an unprecedented dengue outbreak from the year 2014 to 2016 that resulted in an enormous increase in the number of cases and mortality as compared to previous years. The causes that attribute to a dengue outbreak can be multifactorial. Viral factors, such as dengue serotype and genotype, are the components of interest in this study. Although only a small number of studies investigated the association between the serotype of dengue virus and clinical manifestations, none of these studies included analyses on dengue genotypes. The present study aims to investigate dengue serotype and genotype-specific clinical characteristics among dengue fever and severe dengue cases from two Malaysian tertiary hospitals between 2014 and mid-2017.

**Methodology and principal findings:**

A total of 120 retrospective dengue serum specimens were subjected to serotyping and genotyping by Taqman Real-Time RT-PCR, sequencing and phylogenetic analysis. Subsequently, the dengue serotype and genotype data were statistically analyzed for 101 of 120 corresponding patients’ clinical manifestations to generate a descriptive relation between the genetic components and clinical outcomes of dengue infected patients. During the study period, predominant dengue serotype and genotype were found to be DENV 1 genotype I. Additionally, non-severe clinical manifestations were commonly observed in patients infected with DENV 1 and DENV 3. Meanwhile, patients with DENV 2 infection showed significant warning signs and developed severe dengue (p = 0.007). Cases infected with DENV 2 were also commonly presented with persistent vomiting (p = 0.010), epigastric pain (p = 0.018), plasma leakage (p = 0.004) and shock (p = 0.038). Moreover, myalgia and arthralgia were highly prevalent among DENV 3 infection (p = 0.015; p = 0.014). The comparison of genotype-specific clinical manifestations showed that DENV 2 Cosmopolitan was significantly common among severe dengue patients. An association was also found between genotype I of DENV 3 and myalgia. In a similar vein, genotype III of DENV 3 was significantly common among patients with arthralgia.

**Conclusion:**

The current data contended that different dengue serotype and genotype had caused distinct clinical characteristics in infected patients.

## Author summary

The study highlights interesting relationship between viral factors and clinical manifestation of dengue disease during an outbreak. The viral factors which include serotype and genotype of dengue virus were studied to discover if the clinical manifestation in patients were serotype and genotype-specific. As most clinical symptoms of severe dengue infection only manifest at a much later stage of dengue infection, therefore, information on serotype or genotype-specific dengue manifestations may serve as early surrogate markers to predict disease progression. We found that specific clinical manifestations were over-represented by a specific DENV serotype and genotype. Severe dengue was significantly present in DENV 2 Cosmopolitan-infected group while non-severe dengue was prominent among DENV 1 genotype I-infected patients. DENV 3-infected patients commonly manifested musculoskeletal symptoms. This study was undertaken between 2014 and mid-2017, which was considered to be crucial, given that Malaysia experienced an unprecedented outbreak of dengue during this period. Consequently, the occurrence of serotype shift and possible re-emergence of DENV 3 genotype I were reported.

## Introduction

Since the 1950’s, dengue has become a serious health problem in the South-East Asia region. In 1902, Malaysia experienced its first case of dengue [1]. Since then, Malaysia has increasingly become popular for perpetual dengue endemic issues, resulting from the continuous rise in reported dengue infection cases. The country experienced major outbreaks in 1974, 1978, 1982, 1990. Notably, Malaysia recorded the highest number of dengue cases between 2014 and 2017. In 2014, a total of 108,698 cases were reported in Malaysia which was equivalent to an incidence rate (IR) of 361.1 cases in 100,000 populations with 215 mortalities. This alarming figure had outnumbered the previous recorded dengue cases in 2013 by 150.8%. Meanwhile, there was a noticeable increase in 2015 with 120,836 cases (IR = 396.4), along with 336 mortalities. In 2016, the total number of dengue cases declined to around 101,357, although, the mortality rate was 10% higher as compared to that in 2014. [2, 3]. Finally in 2017, the dengue situation in Malaysia came under control as the total number of cases continued to drop. However, the number was still higher as compared to that in 2013. Subsequently, this prompted the Malaysian government to implement a program to eradicate *Aedes aegypti* mosquito breeding sites. Recently, a National Dengue Plan (2015–2020) was implemented by the Malaysian government to intensify the readiness and response capacity in detecting dengue cases and outbreaks, requiring immediate action and attention.

Several risk factors at various intensities have contributed towards the severity of dengue infection during the course of an outbreak. Viral factors are often considered as one of the risk factors and components of interest in many studies in this field. A shift in the distribution of dengue serotypes and genotypes may contribute to the accelerating number of dengue cases due to an antibody-dependent enhancement (ADE) effect [4]. Interestingly, new genotype clades were discovered in some countries such as India and Sri Lanka during dengue outbreaks [5, 6]. While many studies investigated the association between certain serotype of dengue virus and disease severity, only a few studies provided comparative details of the clinical manifestations among dengue serotypes and genotypes [7, 8, 9]. This comparison is particularly important to further aid in the early prediction of a patient’s condition based on the clinical characteristics and information on the serotype and genotype of the dengue virus infecting the patient.

With advanced laboratory tests, the serotyping of the dengue virus from an infected patient can be performed even on the first day of fever. Most clinical symptoms of severe dengue infection only manifest at a later stage of dengue infection. Therefore, information on serotype or genotype-specific dengue manifestations may serve as early surrogate markers to predict disease progression. Furthermore, specific clinical manifestations may be over-represented in patients infected with certain DENV serotype and genotype. In consideration of the above discussion, this study aims to investigate the clinical manifestations of dengue patients in relation to dengue serotype and genotype during a dengue outbreak period in Malaysia.

## Materials and methods

### Ethics statement

This study has obtained an ethical approval from the Medical Research & Ethics Committee, Ministry of Health Malaysia (Reference number: NMRR-15-923-25233). All patients’ data were totally anonymous and requested from clinicians involved in this study.

### Study setting and design

This is a retrospective observational study performed with a total of 120 dengue serum specimens obtained from two tertiary hospitals and a research institute in Malaysia. The serum specimens were acquired from patients who were primarily admitted for dengue fever and confirmed for dengue infection at Hospital Serdang (n = 94) and Hospital Ampang (n = 24) from October 2014—May 2017. Two more sera from DENV 4-infected patients were obtained from the Virology Unit, at the Institute for Medical Research (IMR), in Kuala Lumpur. The inclusion criteria at the time of sample collection included samples that were positive for dengue NS1 antigen whereas exclusion criteria included suspicion for dengue but were negative for NS1 and identified as other febrile illnesses. The dengue confirmation criteria incorporated the results from the NS1 antigen rapid test, with or without Dengue IgM/IgG rapid combo tests performed by the hospitals. These tests were typically performed as soon as dengue infection was suspected in a patient. If dengue IgM was negative before day seven of the onset of fever, a repeat sample was taken at the recovery phase. The IgM and IgG rapid tests results were used to classify the cases as primary and secondary infection. Dengue cases that were positive for IgM but negative for IgG were regarded as primary infection whereas cases that were positive for either IgG only or both IgM and IgG were classified as secondary infection. The commercially available diagnostic kits incorporate an immunochromatography-based technique manufactured by Pan Bio (Brisbane, Australia). The NS1 antigen rapid test was interpreted by a single band targeting the dengue NS1 antigen. The IgM/IgG Dengue Duo Cassette highlights the presence of anti-dengue IgM and IgG antibodies in their specific bands. The results of the tests were displayed as reactive or non-reactive without titration. The reactive IgG result was semi-quantitative, showing the presence of antibodies in the serum which was equivalent to HAI titer of 1:2,560, indicating a secondary dengue infection.

Dengue fever was diagnosed and defined according to the World Health Organization (WHO) 2009 dengue classification and severity level. The classification was used to indicate dengue, dengue with warning sign and severe dengue. Severe dengue includes severe plasma leakage, severe haemorrhage and severe organ dysfunction. To make the clinical diagnosis and determine the severity of the dengue infection, a medical officer performed physical examination on the patient, after which the findings were keyed in the e-file and request was made for necessary laboratory tests to be performed. Following this, a team of experienced clinicians including consultants or specialists further verified the accuracy of the clinical diagnosis during daily clinical ward round based on patients’ progress in their symptoms, clinical findings as well as the latest laboratory test report. The implication of the observation was to determine whether the patient requires intensive unit (ICU) care or normal ward management.

Dengue serotyping and phylogenetic analysis were performed for all 120 specimens. Socio-demographic, clinical profiles and laboratory data were obtained from the patient's record from the respective hospitals. The information was later analyzed with the dengue serotyping and genotyping results performed in this study. Notably, in the sta-tistical analysis, some samples were excluded due to incomplete clinical information, the presence of co-infection with leptospirosis and small sample size for a particular serotype. The rationale for excluding the samples of patients co-infected with leptospirosis was due to the overlapping clinical features of the patients infected with leptospirosis and dengue. These samples, however, were not tested for other closely related co-infections such as Zika or Chikungunya due to budgetary constraints. Moreover, the information on the history and clinical presentation of the patients were unlikely to be of these diseases.

### Dengue viral RNA extraction

QIAamp Viral RNA Mini Kit (Qiagen) was used to perform the extraction of dengue viral ribonucleic acid (RNA) from the serum specimens according to the manufacturer’s instructions. The eluted 50 μl viral RNA was used as a template in the PCR assays.

### Serotyping by fourplex Taqman Real-Time RT-PCR

Dengue virus serotyping was carried out in a fourplex Taqman Real-Time RT-PCR detection platform as described by Johnson *et al*. (2005) [10]. PCR reactions were prepared in a cocktail of 12.5 μl of 2X RT (reverse transcriptase)-PCR Mix (i-Script One Step RT-PCR kit, Biorad, USA), 0.5 μl of each primers (DENV 1 and DENV 3 primers: 50 μM; DENV 2 and DENV 4 primers: 25 μM), 0.45 μl of each probes (10μM), 0.5 μl of RT Enzyme Mix, and 1.2 μl of nuclease-free water. Positive controls for each serotype comprised of RNA from previously confirmed dengue patients, and obtained from the Virology Unit, IMR. The negative control consisted of reactions without an RNA template, which was substituted with 5μl of nuclease-free water. Taqman Real-Time RT-PCR amplification was performed on the CFX 96 (Biorad, USA) platform at 50°C for 10min, 95°C for 5min, followed by 45 cycles of 95°C for 15 sec and 60°C for 30 sec. The aforementioned PCR mix and cycling conditions were optimized by the Virology Unit. io: dx.doi.org/10.17504/protocols.io.rabd2an.[PROTOCOL DOI]

### Serotype-specific PCR amplification and sequencing

The partial E gene of dengue virus was amplified before sequencing by using four sets of serotype-specific oligonucleotides [11]. All amplification reactions were carried out in a 96-well conventional Thermal Cycler (Bio Rad, USA). The PCR was undertaken at 50°C for 30 min, 94°C for 2 min and 45 cycles of (94°C for 15 sec, 50°C for 30 sec and 68°C for 1 min) followed by an extension reaction at 68°C for 5 min. A 25 μl aliquot of each PCR reaction was analyzed on 1.5% pre-stained agarose by gel electrophoresis and viewed under UV illumination. The corresponding amplicons were extracted from the agarose gel and purified by a Gel Extraction Kit (Qiagen, USA) according to the manufacturer’s instruction. The final elution contained 30 μl of purified PCR amplicons whereby 5 μl of these were reanalyzed on 1.5% agarose gel to substantiate the accuracy of purification step. The purified PCR amplicons were outsourced for Sanger Sequencing (1^st^ Base, IDT, Singapore). io:dx.doi.org/10.17504/protocols.io.racd2aw.[PROTOCOL DOI]

### Genotyping by phylogenetic analysis

The sense and antisense sequences obtained by sequencing were aligned to produce a consensus partial E gene sequence by using CLUSTAL Omega software (https://www.ebi.ac.uk/Tools/msa/clustalo/). Reference sequences of E gene for each serotype were extracted from the GenBank database from various geographical regions. Phylogenetic trees were constructed with Mega 7 software adopting neighbor-joining method (bootstrap replication 1000x) for all four serotypes to determine the genotypes of dengue virus isolates.

### Statistical analysis

Statistical package for the social sciences (SPSS) version 21.0 was adopted to analyze data collected from the dengue patients. Categorical variables were expressed as frequencies (percentages). Chi-square or Fisher’s Exact test was performed to analyze the significance of the categorical variables. Continuous variables were tested for normality with the Komolgorov-Smirnov test. Non-parametric analysis by Kruskal-Wallis was employed for data with non-normal distribution and presented in median and interquartile range (IQR). The normally distributed data were analyzed by One-Way ANOVA and expressed as mean and standard deviation (SD). The patients’ data were tabulated according to categorical variables including gender, serotypes, genotypes and a spectrum of clinical manifestations. Meanwhile, continuous variables refer to parameters such as age, day of fever and laboratory test results. These data were utilized to determine the distribution of dengue serotype and genotype in the study population and describe the relation with demography, clinical manifestations and laboratory parameters. The analyses were performed at 95% confidence with level of significance of p<0.05.

## Results

### Distribution of dengue serotype and genotype

**Fig 1:**
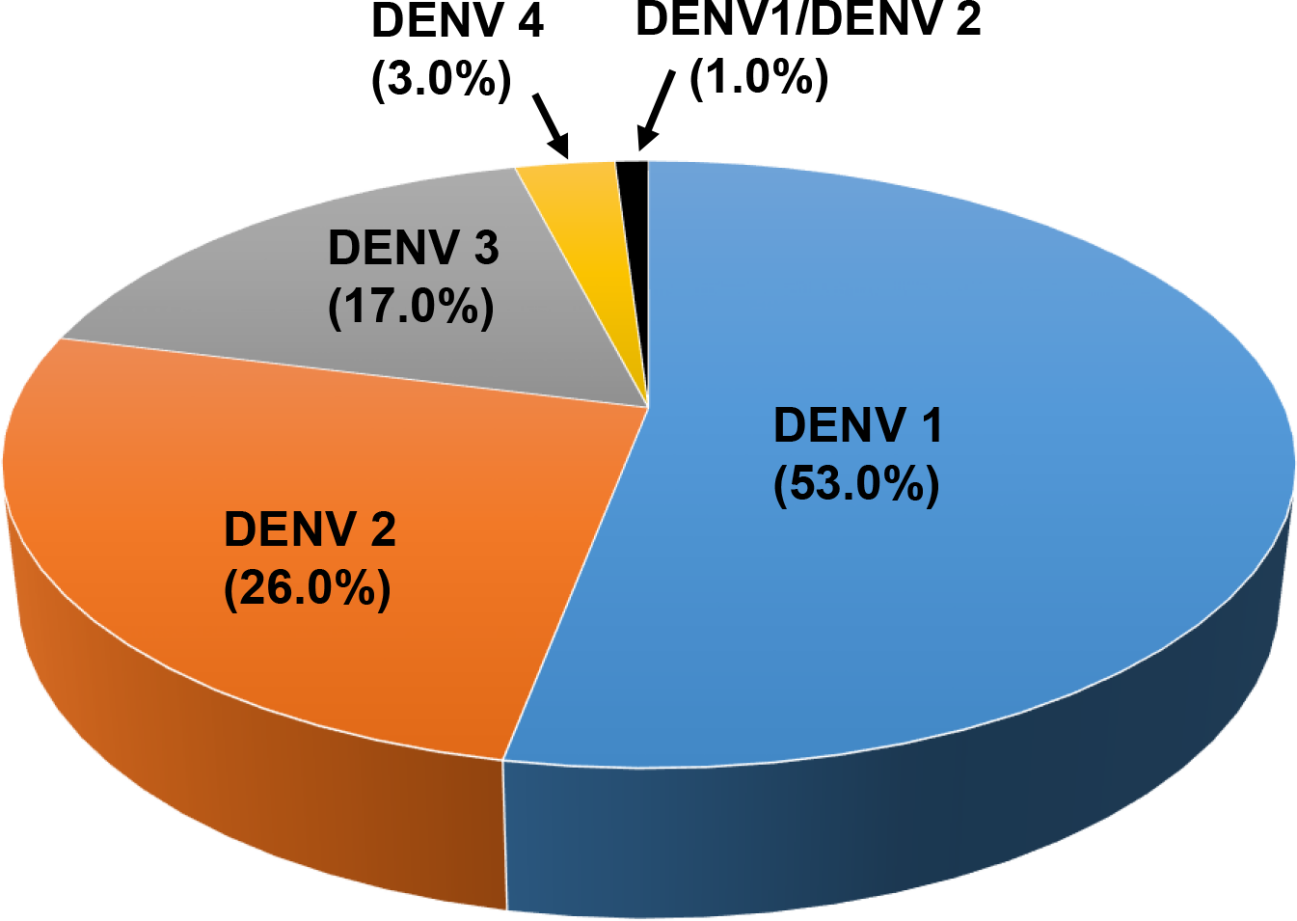
Distribution of dengue cases by serotypes among the study population.

**Fig 2:**
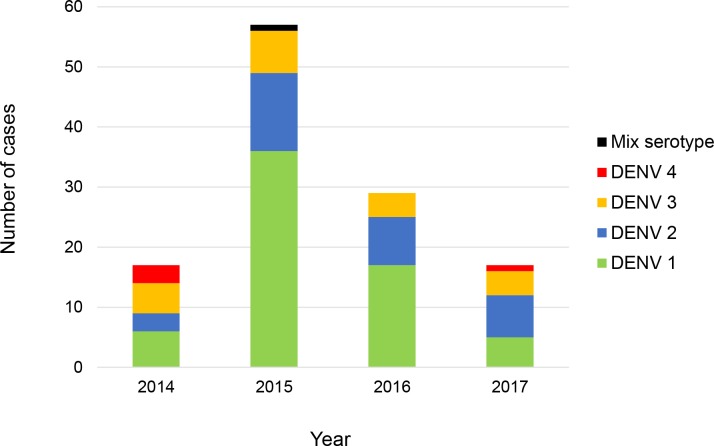
Distribution of dengue serotypes by year of infection among the study population.

**Fig 3:**
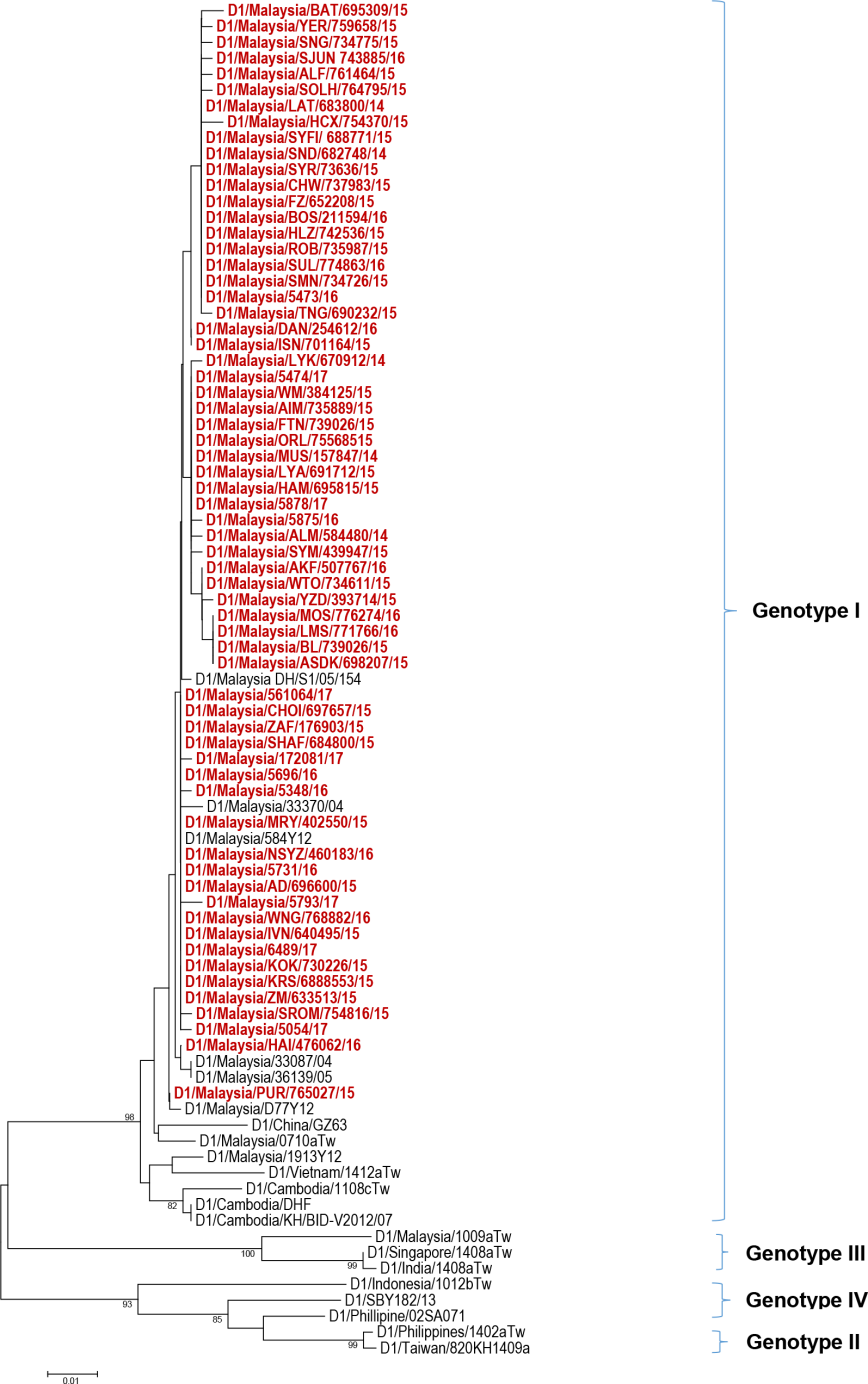
Phylogeny of DENV 1 virus based on E gene sequence. Dengue isolates from the study are represented in red.

**Fig 4:**
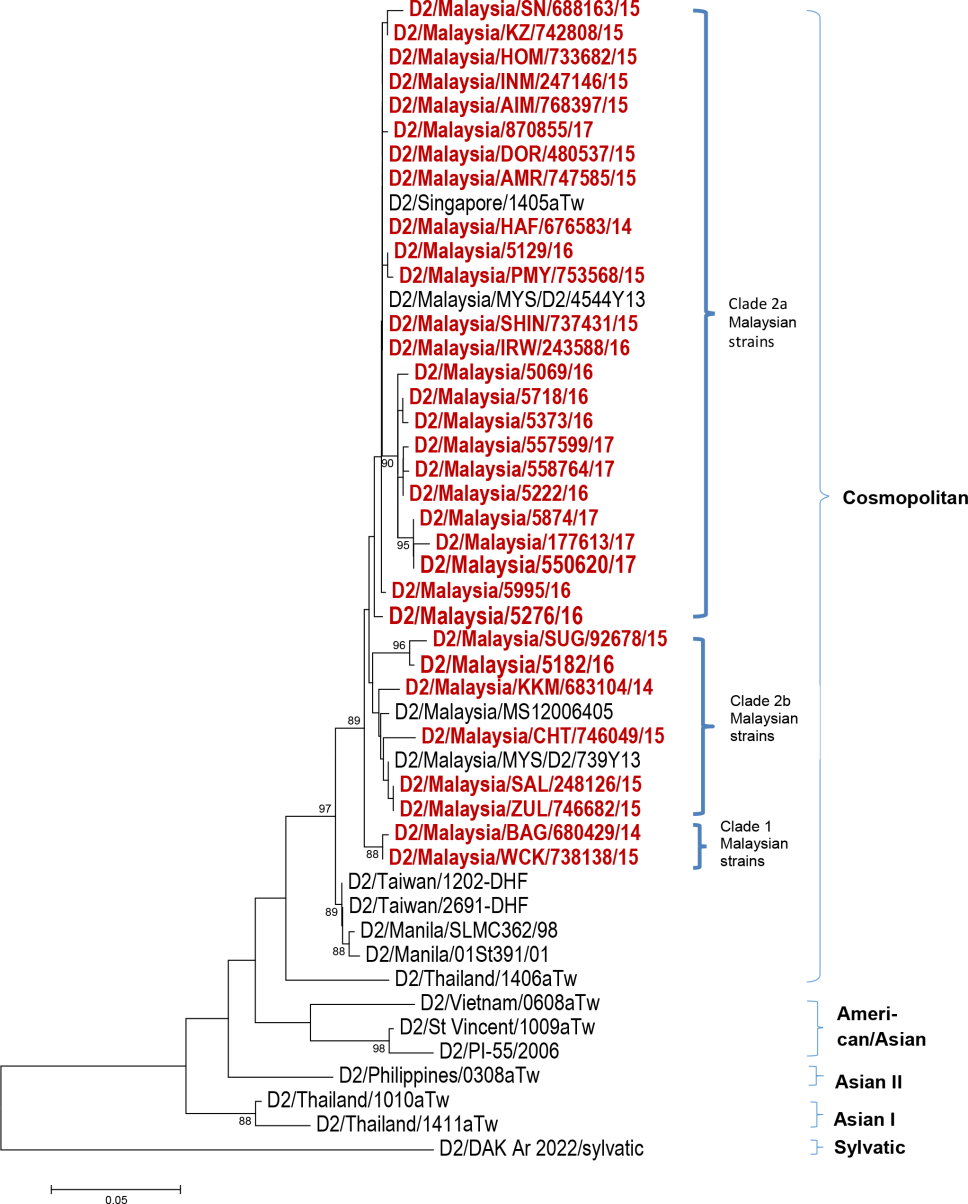
Phylogeny of DENV 2 virus based on E gene sequence. Dengue isolates from the study are represented in red.

**Fig 5:**
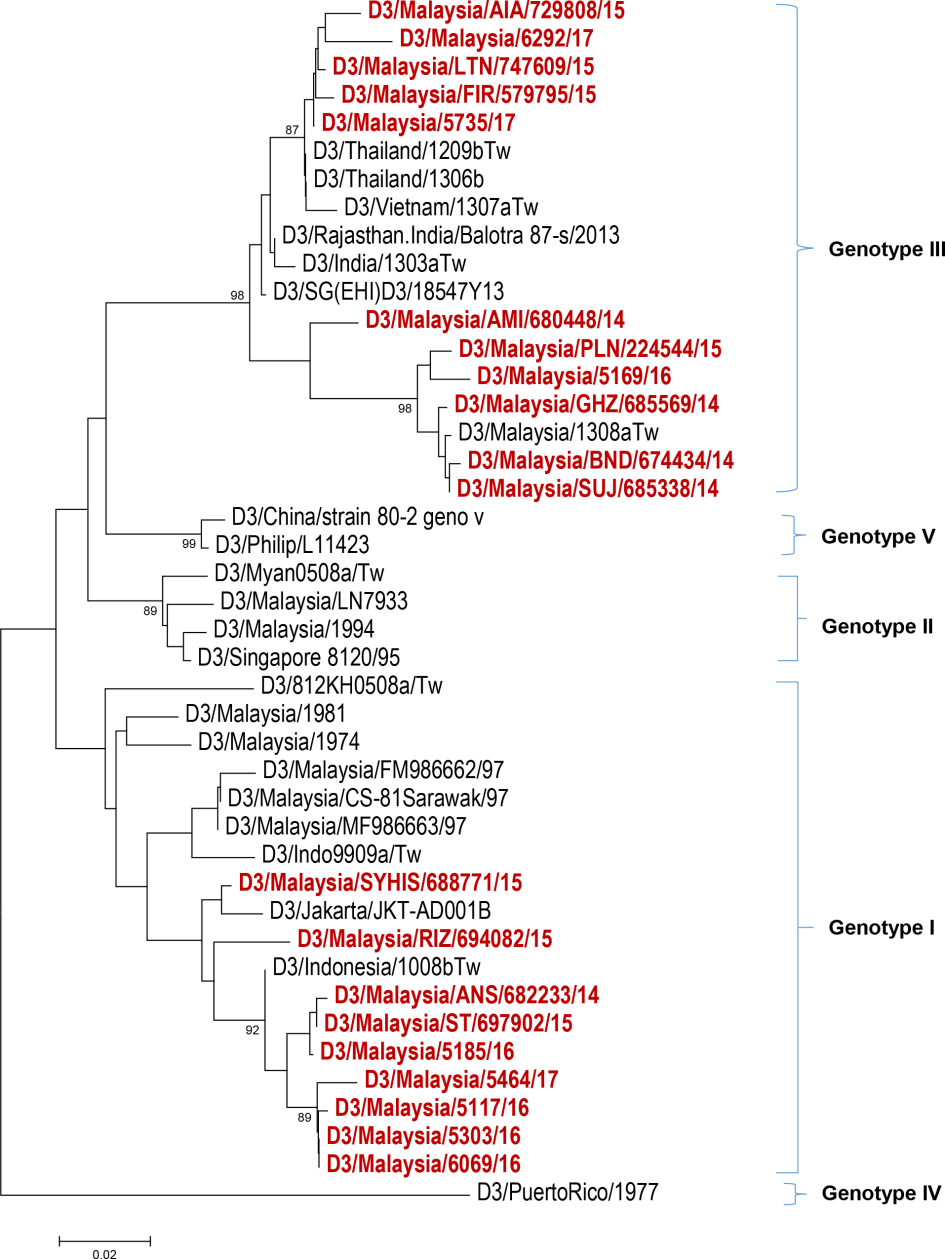
Phylogeny of DENV 3 virus based on E gene sequence. Dengue isolates from the study are represented in red.

**Fig 6:**
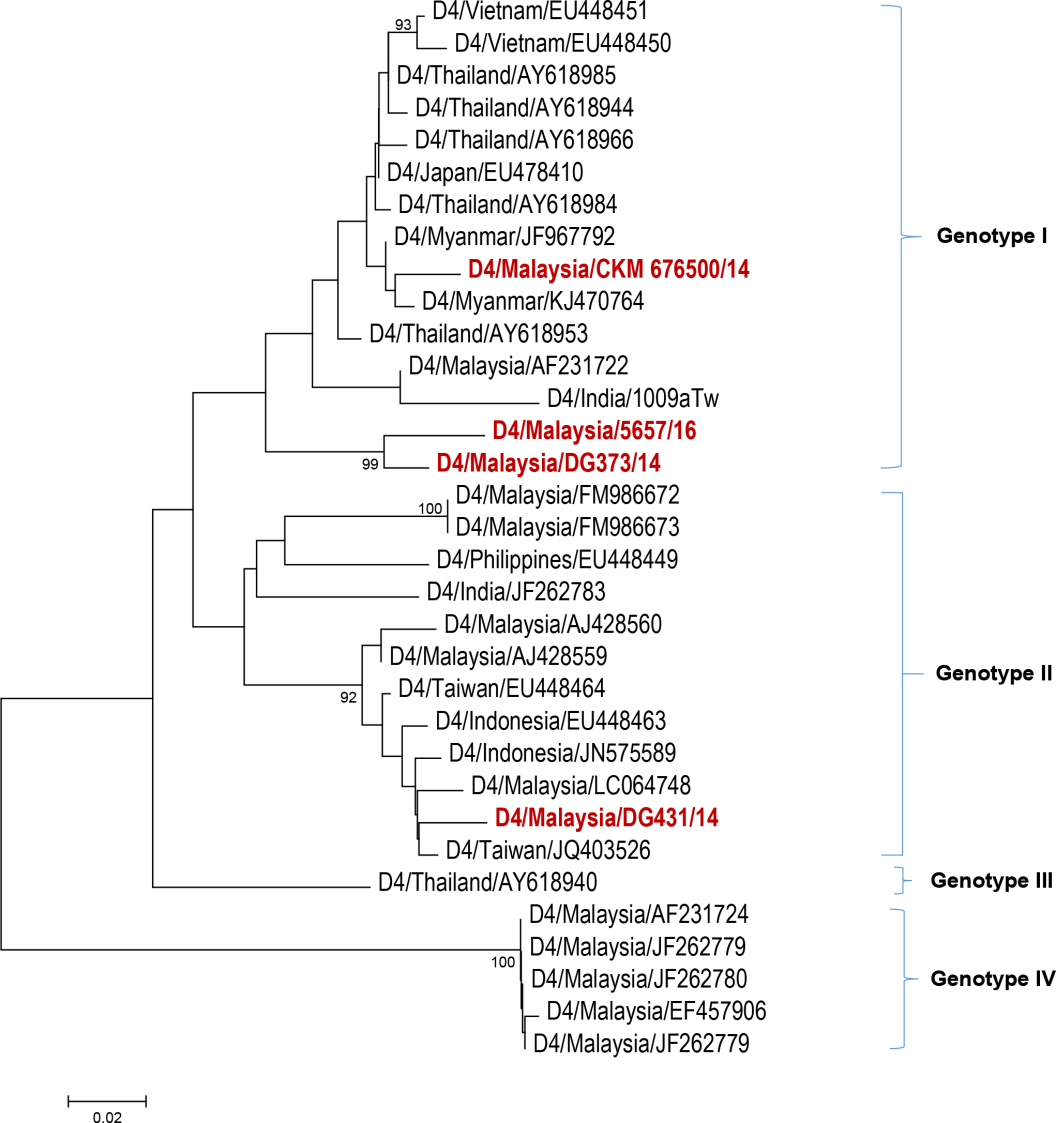
Phylogeny of DENV 4 virus based on E gene sequence. Dengue isolates from the study are represented in red.

Serotyping results (Fig 1) from 120 study subjects revealed that more than half of the study population were infected with DENV 1 (64/120; 53.0%) followed by DENV 2 (31/120; 26.0%), DENV 3 (20/120; 17.0%), DENV 4 (4/120; 3.0%) and mixed serotype DENV 1/ DENV 2 (1/120; 1.0%). The distribution of these serotypes by year of infection is shown in Fig 2. Among our study subjects, a domination of DENV 1 was seen from the year 2014–2016 with prevalence of 35.3% (6/17), 63.2% (36/57) and 58.6% (17/29) each year, respectively. In 2017, DENV 2 was more frequently observed than other serotypes (7/17; 41.2%) among the study subjects. The number of DENV 3-infected cases from 2014–2017 were less than DENV 1 and DENV 2 except in the year 2014 with prevalence of 29.4% (5/17), 19.4% (7/57), 13.8% (4/9) and 23.5% (4/17), respectively. DENV 4-infected cases were observed more in 2014 (3/17; 17.6%) and one case in 2017 (1/17; 5.9%) while one DENV 1/ DENV 2 mix serotype case was found in 2015 (1/17; 5.9%). Further, phylogenetic analysis classified these dengue strains into genotypes (Figs 3–6). All DENV 1 and DENV 2 strains were classified under genotype I and cosmopolitan genotype, respectively. DENV 3 strains clustered into two distinct genotypes. Genotype III comprised most of the DENV 3 strains as compared to genotype I. The DENV 4 strains from the study belong to genotype I and genotype II. In addition, one dengue strain with mixed serotypes of DENV 1/DENV 2 were identified. However, only DENV 2 from the mixed strain could be amplified by PCR and sequenced. All 120 sequences were deposited in NCBI with accession numbers MG450795 –MG450914.

### Phylogeny analysis

The 2014–2017 DENV 1 strains (Fig 2) displayed a monophyletic relationship being clustered in the genotype I group. These strains showed a distant connection with strains from Vietnam, Cambodia, China and Taiwan-imported case from the same genotype. D1/Malaysia/330877/04 and D1/Malaysia/36139/05, which were two strains from the former 2004–2005 outbreaks formed their own clade, quite distinctively from the recent outbreak strains. One recent outbreak strain (D1/Malaysia/PUR/765027/15), isolated in December 2015 was noticeably set apart from other DENV 1 genotype I strains. There was no obvious clustering within the 2014–2017 DENV 1 strains as all of them were randomly dispersed within the clades.

The DENV 2 phylogeny (Fig 3) displayed a well-defined clade formation within the Cosmopolitan genotype. The Cosmopolitan genotype of Malaysian strains formed two main clades, which are referred to as Clade 1 and Clade 2. Clade 2 was further divided into sub-clade 2a and sub-clade 2b. There were two strains within Clade 1, namely D2/Malaysia/BAG/ 680429/14 and D2/Malaysia/WCK/738138/15, which were isolated in December 2014 and August 2015, respectively. Clade 2 comprised of strains isolated during the 2014–2017 outbreak. Domination of dengue strains by year of outbreak within sub-clade 2a and sub-clade 2b was observed. Early outbreak strains from the year 2014–2015 clustered in sub-clade 2b whereas late outbreak strains from the year 2015–2017 dominated sub-clade 2a.

The DENV 3 phylogeny (Fig 4) classified the current outbreak strains into genotype I and genotype III. The 2014–2015 DENV 3 strains were commonly noticed among genotype III clade, whereas the 2016–2017 strains appeared more in genotype I clades. The dengue strains from genotype III branched into two clades whereas genotype I strains were clustered together. The DENV 4 strains in this study belonged to genotype I and genotype II (Fig 5).

### Dengue serotype and genotype and clinical manifestations of infected patients

Of the total 120 cases investigated, 101 cases were included in the statistical analysis while the remaining 19 were omitted. As mentioned earlier, the excluded cases were those with incomplete clinical information, co-infection with leptospirosis and small sample size for particular serotype (DENV 4 and mixed serotype). The demographic details of the 101 study subjects are shown in Table 1. Age of the patients was significantly related to dengue serotypes. The median age group for DENV 1, DENV 2 and DENV 3-infected patients was 14, 27 and 23 respectively. A significant relationship was detected between dengue serotypes and disease classification (p = 0.007) (Table 2). Dengue infections without warning signs were observed more frequently in patients who were infected with DENV 1 (29/58; 50.0%) and DENV 3 (8/16; 50.0%). Notably, DENV 2-infected patients more frequently developed severe dengue (9/27; 33.3%). In the present study, a total of 17 severe dengue cases were identified of which two were noted as fatal cases. Apart from this, dengue with warning signs were frequently displayed by patients infected with DENV 2 (13/27; 48.1%).

**Table 1:** Demographic details of study subject.

Demographic data	Total subject (n = 101)	DENV 1 (n = 58)	DENV 2 (n = 27)	DENV 3 (n = 16)	P Value
**Days of fever, median days (IQR)**	4 (3–5)	4 (3–5)	3 (3–4)	4 (3–4)	0.918
**Age, median years (IQR)**	20 (12–30)	14 (9–26)	27 (17–39)	23 (17–32)	0.001*

The total n = 101 reflects the total subjects included in the statistical analysis after the exclusion criteria was taken into consideration.

* Indicates a significant result with P value < 0.05

**Table 2:** Dengue serotypes and genotypes and clinical manifestations of dengue patients.

Parameters	DENV 1	DENV 2	DENV 3	P value	Geno I	Cosmo	Geno I	Geno III	P value
	(n = 58)	(n = 27)	(n = 16)		DENV 1	DENV 2	DENV 3	DENV 3	
					(n = 58)	(n = 27)	(n = 7)	(n = 9)	
	% (n)	% (n)	% (n)		% (n)	% (n)	% (n)	% (n)	
**Disease category**				0.007*					0.025*
With warning signs	39.7 (23)	48.1 (13)	37.5 (6)		39.7 (23)	48.1 (13)	57.1 (4)	22.2 (2)	
Without warning signs	50.0 (29)	18.5 (5)	50.0 (8)		50.0 (29)	18.5 (5)	42.9 (3)	55.6 (5)	
Severe dengue	10.3 (6)	33.3 (9)	12.5 (2)		10.3 (6)	33.3 (9)	0.0 (0)	22.2 (2)	
**Specific manifestations**									
**• With warning signs**									
Lethargy	15.5 (9)	14.8 (4)	12.5 (2)	0.956	15.5 (9)	14.8 (4)	14.3 (1)	11.1 (1)	0.989
Persist vomiting	29.3 (17)	63.0 (17)	50.0 (8)	0.010*	29.3 (17)	63.0 (17)	57.1 (4)	44.4 (4)	0.024*
Epistaxis	3.4 (2)	0.0 (0)	6.3 (1)	0.480	3.4 (2)	14.3 (1)	0.0 (0)	0.0 (0)	0.235
Epigastric pain	12.1 (7)	37.0 (10)	12.5 (2)	0.018*	12.1 (7)	37.0 (10)	14.3 (1)	11.1 (1)	0.045*
Abdominal pain	19.0 (11)	22.2 (6)	25.0 (4)	0.851	19.0 (11)	22.2 (6)	42.9 (3)	11.1 (1)	0.435
Ascites	6.9 (4)	11.1 (3)	0.0 (0)	0.382	6.9 (2)	11.1 (1)	0.0 (0)	0.0 (0)	0.589
Gum bleed	3.4 (2)	3.7 (1)	12.5 (2)	0.316	3.4 (2)	3.7 (1)	14.3 (1)	11.1 (1)	0.496
Diarrhea >3x a day	20.7 (12)	14.8 (4)	12.5 (2)	0.670	20.7 (12)	14.8 (4)	0.0 (0)	22.2 (2)	0.546
**• Without warning signs**									
Headache	29.3 (17)	37.0 (10)	37.5 (6)	0.704	29.3 (17)	37.0 (10)	42.9 (3)	33.3 (3)	0.834
Retro orbital pain	6.9 (4)	11.1 (3)	0.0 (0)	0.382	6.9 (4)	11.1 (3)	0.0 (0)	0.0 (0)	0.589
Chills	22.4 (13)	25.9 (7)	25.0 (4)	0.932	22.4 (13)	25.9 (7)	28.6 (2)	22.2 (2)	0.973
Rigor	20.7 (12)	22.2 (6)	25.0 (4)	0.932	20.7 (12)	22.2 (6)	28.6 (2)	22.2 (2)	0.972
Cough	8.6 (5)	22.2 (6)	25.0 (4)	0.147	8.6 (5)	22.2 (6)	0.0 (0)	11.1 (1)	0.231
Sore throat	3.4 (2)	14.8 (4)	6.3 (1)	0.065	3.4 (2)	14.8 (4)	0.0 (0)	0.0 (0)	0.141
Rhinorrhea	6.9 (4)	7.4 (2)	0.0 (0)	0.990	6.9 (4)	7.4 (2)	0.0 (0)	11.1 (1)	0.856
Myalgia	43.1 (25)	63.0 (17)	81.3 (13)	0.015*	43.1 (25)	63.0 (17)	85.7 (6)	77.8 (7)	0.036*
Arthralgia	36.2 (21)	55.6 (15)	75.0 (11)	0.014*	36.2 (21)	55.6 (15)	71.4 (5)	77.8 (7)	0.035*
Rash	8.6 (5)	11.1 (3)	0.0 (0)	0.408	8.6 (5)	11.1 (3)	0.0 (0)	0.0 (0)	0.617
**• Severe dengue**									
Severe bleeding	12.1 (7)	7.4 (2)	0.0 (0)	0.308	12.1 (7)	7.4 (2)	0.0 (0)	0.0 (0)	0.502
Severe organ failure	3.4 (2)	11.1 (3)	6.3 (1)	0.379	3.4 (2)	11.1 (3)	0.0 (0)	11.1 (1)	0.422
Shock	10.3 (6)	29.6 (8)	6.3 (1)	0.038*	10.3 (6)	29.6 (8)	0.0 (0)	11.1 (1)	0.075
Severe Plasma leakage	5.2 (3)	25.9 (7)	0.0 (0)	0.004*	5.2 (3)	25.9 (7)	0.0 (0)	0.0 (0)	0.012*

* Indicates a significant result with P value < 0.05

The investigation of dengue serotype-specific clinical manifestations demonstrated that DENV 2-infected patients were more frequently present with persistent vomiting (p = 0.010), epigastric pain (p = 0.018), severe plasma leakage (p = 0.004) and shock (p = 0.038). Additionally, myalgia and arthralgia were the two major musculoskeletal symptoms observed in DENV 3 infection (p = 0.015, p = 0.014). Although statistically insignificant, relatively high proportion of DENV 1-infected patients suffered from lethargy (9/58; 15.5%) and diarrhea (12/58; 20.7%). Furthermore, genotype I of DENV 3 was frequently found in patients with myalgia (p = 0.036). Likewise, genotype III of DENV 3 and arthralgia were found to be associated with one another (p = 0.035).

### Laboratory parameters in relation to dengue serotype and genotype

Investigation of laboratory parameters in relation to dengue serotype and genotype is shown in Table 3. None of the laboratory parameters were significant among dengue serotypes and genotypes. A majority of the study subjects that were infected with DENV 1 (57/68; 98.3%) and DENV 2 (26/27; 96.3%) had primary infection. The percentage of secondary infection was greater in the DENV 3 (2/16; 12.5%) infected group than other serotypes. The mean platelet count was the lowest among DENV 2-infected patients as indicated by mean (±SD) of 106 x 10^9^ (±71.5) whereas the hematocrit level was the highest among DENV 3 patients (43.0 (±5.9)).

**Table 3:** Dengue serotypes and genotypes and laboratory parameters.

Laboratory parameters	DENV 1 (n = 58)	DENV 2 (n = 27)	DENV 3 (n = 16)	P value	Geno I DENV1 (n = 58)	Cosmo DENV 2 (n = 27)	Geno I DENV 3 (n = 7)	Geno III DENV 3 (n = 9)	P value
									
**Infection status**				0.147					0.268
Primary infection, % (n)	98.3 (57)	96.3 (26)	87.5 (14)		98.3 (57)	96.3 (26)	85.7 (6)	88.9 (8)	
Secondary infection, % (n)	1.7 (1)	3.7 (1)	12.5 (2)		2.0 (1)	3.7 (1)	14.3 (1)	12.5 (1)	
**Platelet count** (x 10^9^), mean (±SD)	148.1 (±74.0)	106.0 (±71.5)	135.2 (±54.6)	0.079	148.1 (±74.0)	106.0 (±71.5)	119.7 (±61.8)	147.2 (±67.8)	0.131
**Hematocrit level** (%), mean (±SD)	40.1 (±3.6)	41.6 (±4.1)	43.0 (±5.9)	0.061	40.1 (±3.6)	41.6 (±4.1)	42.3 (±5.3)	43.5 (±6.5)	0.085
**White blood cell** (x 10^9^), median (IQR)	3.8 (2.6–5.4)	3.2 (1.9–4.1)	3.7 (2.8–4.8)	0.145	3.8 (2.6–5.4)	3.2 (1.9–4.1)	3.3 (2.6–5.6)	3.7 (2.9–4.5)	0.275
**AST** (IU/ml), median (IQR)	53.0 (30.0–155.0)	83.0 (34.3–248.0)	55.5 (33.3–124.0)	0.683	53.0 (30.0–155.0)	83.0 (34.3–248.0)	49.0 (35.5–84.5)	99.0 (31.0–180.0)	0.671
**ALT** (IU/ml), median (IQR)	45.0 (23.0–110.0)	67.5 (22.8–164.8)	53.0 (19.0–96.5)	0.651	45.0 (23.0–110.0)	67.5 (22.8–164.8)	27.0 (19.0–54.5)	84.0 (24.5–125.0)	0.444

Serotype association with disease severity in regards to primary and secondary infection was elucidated in Table 4. In the primary infection group, the percentage of severe and non-severe dengue cases was 16.5% (16/97) and 83.5% (81/97), respectively. Also, within the primary infection group, disease severity was significantly associated with dengue serotype (p = 0.014). Among the severe dengue cases within the primary infection group, a great proportion were infected with DENV 2 (9/16; 56.3%) followed by DENV 1 (6/16; 37.5%) and DENV 3 (1/16; 6.3%) whereas among the non-severe dengue cases, 63.0% (51/81) were infected with DENV 1, followed by DENV 2 (17/81; 21.0%) and DENV 3 (13/81; 16.0%). In addition, within the secondary infection group, the percentage of severe cases was 25.0% (1/4) while 75.0% (3/4) were non-severe cases. The only severe dengue case in the secondary infection group was infected with DENV 3 while the three non-severe cases were each infected with DENV 1, DENV 2, and DENV 3.

**Table 4:** Dengue severity among primary and secondary infection in relation with serotypes.

Infection type	Severity	DENV 1 (n = 58)	DENV 2 (n = 27)	DENV 3 (n = 16)	P value
Primary infection (n = 97)	Severe dengue (n = 16) % (n)	37.5 (6)	56.3 (9)	6.3 (1)	0.014*
	Non-severe dengue (n = 81) % (n)	63.0 (51)	21.0 (17)	16.0 (13)	
Secondary infection (n = 4)	Severe dengue (n = 1) % (n)	0 (0)	0 (0)	100.0 (1)	0.161
	Non-severe dengue (n = 3) % (n)	33.3 (1)	33.3 (1)	100.0 (1)	

* Indicates a significant result with P value < 0.05

## Discussion

The present study focused on identifying the pattern of dengue serotype and genotype distribution from the year 2014–2017 in Malaysia and attempted to investigate the clinical spectrum of patients in relation to this distribution pattern. It was found that DENV 1 genotype I was the predominant serotype and genotype in the recent dengue outbreak in Malaysia. This reflected a serotype shift replacing the formerly predominant DENV 2 prior to the outbreak. Comparison of dengue serotype and genotype with disease spectrum contended that these clinical characteristics are serotype and genotype-specific. As most clinical symptoms of severe dengue infection only manifest at a much later stage of dengue infection, therefore, information on serotype or genotype-specific dengue manifestations may serve as early surrogate markers to predict disease progression.

In Malaysia, the serotype distribution of dengue virus has been inconsistent. However, there was a seemingly interesting pattern of circulation during outbreak period whereby major outbreaks were likely to follow the switching of DENV serotypes [12]. A predominantly circulating dengue serotype before an outbreak is replaced by another serotype which persists towards the end of the outbreak. Once the number of cases decline, the persistent serotype is again replaced by another serotype. For instance, during the 1996–1998 dengue outbreaks in Malaysia, both DENV 1 and DENV 2 dominated other serotypes. Prior to 1996, DENV 3 was consistently circulating. After the outbreak subsided, DENV 2 began to surge from the year 1999 onwards. Similarly, when there was a sharp increase in the number of dengue cases in 2001 and 2002, DENV 2 in the prior years was replaced by DENV 3. Then, DENV 3 was overtaken by DENV 1 from year 2003 onwards as the number of cases declined [2, 13, 14]. In the present study, DENV 1 was primarily present at the study area from 2014–2017. This indicated that DENV 1 had replaced a formerly predominant DENV 2. The existing trend contended that serotype replacement, most probably with DENV 3 is predicted to take place once the current outbreak subsides. In contrast to our prediction, Tan et al (2017) reported the unlikeliness for DENV 3 to surge as serotype cyclical outbreak cycle in Malaysia has recently been disrupted with DENV 3 remained in the background of DENV 1 and DENV 2 from 2003 until now [15].

Apart from serotype replacement, emergence of new genotypes or genotypic clade replacements have been reported during dengue outbreak [16, 17, 18]. This is often attributed to positive pressure selection and viral fitness for survival. In the current study, all DENV 1 strains were classified into genotype I, which subsequently formed minute branches within the same genotype but well conserved. The monophyletic pattern indicated high genetic similarity among the DENV 1 Malaysian strains. As for DENV 2 Cosmopolitan genotype, the current outbreak strains diverged into clades, indicating minor evolution.

The DENV 3 strains in our study were divided into genotype I and genotype III. Genotype III was constantly documented in Malaysia from 2007–2013 [15, 19]. Interestingly, genotype I of DENV 3 was last documented in 2011 and also circulated in this country during dengue epidemics in 1974 and 2007–2008 [15]. Hence, the appearance of genotype I that was equal to genotype III among DENV 3 strains in our study raised the possibility of re-emergence of genotype I especially during outbreaks. DENV 4 is a rare dengue serotype in Malaysia. Three strains from genotype I and a strain from genotype II were detected in the present study. One of the DENV 4 strains clustered together with the dengue strains from Myanmar, reflecting the likelihood of an imported case.

A handful of studies have shown the differences in dengue symptoms are serotype specific, but majority of these studies are conducted outside outbreak periods. A study among adult dengue patients in Singapore from 2005–2011 found that cases infected with DENV 1 were more likely to be presented with red eyes and had higher risk of developing severe dengue. In contrast, those DENV 2-infected patients were found to have frequent joint pains and significantly low platelet count [9]. Another study was conducted among dengue patients in Peru, Bolivia, Ecuador and Paraguay from 2005–2010. The findings showed that DENV 3 had a higher prevalence of musculoskeletal and gastrointestinal manifestations while DENV 4 had a higher prevalence of respiratory and cutaneous manifestations [7]. On the other hand, a group of Indian researchers who undertook a study from 2002–2006 in New Delhi reported a significant result of hepatomegaly and abdominal pain in DENV 2-infected patients. The results of their study also noted that DENV 4 patients suffered from severe hemorrhagic manifestations [20]. All the aforementioned results showed that there were differences in clinical manifestations of dengue patients relating to the serotype owing to the differences in terms of year of study and level of endemicity in particular regions. In response to this, the present study investigated the relationship between dengue serotype and genotype, and the disease spectrum in Malaysia, a dengue hyper endemic country, during a recent outbreak period.

The present study showed a significant number of DENV 2-infected patients developed severe dengue more frequently as compared to other serotypes. These patients were also frequently presented with clinical manifestations such as persistent vomiting, epigastric pain, plasma leakage and shock. Many studies have proven that severity and DENV 2 are common especially in relation to shock manifestation [21, 22, 23]. Similarly in a retrospective study of dengue cases in Thailand, it was also found that DENV 2 was the most frequent serotype isolated from dengue hemorrhagic (DHF) and dengue shock syndrome (DSS) cases followed by DENV 3, DENV 1 and DENV 4 [24]. Although substantial evidence confirmed that DENV 2 caused severe dengue infection, one study found no differences between any DENV serotypes and severity of the disease [25].

Our data suggested that both DENV 1 and DENV 3-infected patients displayed the mildest clinical presentation. The data also signified that DENV 3-infected patients suffered from myalgia and arthralgia. When compared across the genotypes, DENV 2 cosmopolitan was highly prevalent among patients with epigastric pain and shock. Besides that, DENV 3 genotype III was significantly observed among patients with arthralgia whereas DENV 3 genotype I was common among patients with myalgia. Several studies also reported an association between DENV 3 and musculoskeletal manifestations. According to Chan et al. (2009), the findings showed a significant relationship between DENV 3 and myalgia, but the genotype information was unknown [26]. Similarly, Hasley et al (2012) also found that DENV 3-infected patients had frequent myalgia and arthralgia symptoms [7]. Therefore, we hypothesized that DENV 3 has a high preference and binding affinity to the receptors in the musculoskeletal system, narrowing down further that genotype I targets muscles whereas genotype III targets the joints.

The comparison between genotypes also exhibited that genotype III of DENV 3 may appear to be more virulent than genotype I. This is supported by the presence of two severe cases among DENV 3 genotype III-infected patients. These two cases were presented with severe organ impairment and shock (dengue shock syndrome), respectively. This finding may warrant further interest to investigate severity associated with DENV genotype III with inclusion of larger sample size. It can be postulated that since genotype III has been circulating dominantly among DENV 3 genotypes in Malaysia for extended period from its introduction in 2007 till 2013, thus it acquired sufficient survival fitness to become virulent over time as compared to genotype I which was dormant from 2012–2013 and had re-emerged during the outbreak in 2014. However re-emergence of DENV 3 genotype I has to be cautiously monitored as it could undergo adaptation to become more infectious to replace and diminish the existence of DENV 3 genotype III. This highlights the importance of studying serotype and genotype specific clinical manifestations during dengue outbreak to allow prediction of an outbreak outcome.

Previous research had claimed that DENV 1 and DENV 2 are often associated with primary infections and secondary infections, respectively [26]. Contrary to these findings, our investigation revealed insignificant differences between dengue serotypes and infection type as in primary and secondary infection. Indeed, this could be mainly due to low number of secondary infected cases in the present study. In our study, primary dengue infections were found to be more frequent than secondary infections. This finding contradicted with reports of high seroprevalence rate of secondary infection in Malaysia [27]. The seroprevalence rates in Malaysia were shown to be age dependent, whereby as the age increases the seroprevalence also increases and by 70 years old almost 80% of the Malaysian population were already infected with dengue. Our findings on secondary infection rate was different from the above study based on two aspects. Firstly, the distribution of subjects by age group in our study indicate that more than half of the study subjects belonged to younger age group (≤ 20); hence, less likely to have past exposures (S1 Table). Past studies also supported this in which a shift of the dengue infection from affecting primarily children to adults more than age 20 years old, was demonstrated [28, 29]. Secondly, in our study, exclusion of samples with incomplete information, unable to be serotyped or genotyped and the coverage of only two locations contributed to small sample size, thus, we are unable to provide a detailed description of the seroprevalence rate. However, our study findings on high primary infection rate could pose a serious implication if the next outbreak is predominantly caused by DENV 2. It is known that severe dengue is associated with secondary infection and infection with DENV 2 superimposed on previous DENV 1 infection carries the highest risk for development of severe dengue [30]. Even though the DENV 3 upsurge after the current outbreak is predicted, however as mentioned earlier, one study [15] has cautioned that this cycle was recently disrupted, raising the possibility for DENV 2 dominance. Therefore, our study findings on the high prevalence of primary infection is very crucial in the conveyance of early warning for a severe outbreak in the future.

Comparison of serotype severity after stratifying by infection types demonstrated that among primary dengue infection cases, there is a significant association between DENV 2 and the disease severity. This indicates that primary infections caused by DENV 2 may lead to more severe presentations than DENV 1. Interestingly, only one severe case was found in the secondary infection group and no significance was observed among the serotypes and severity in this group. One possible explanation, in this case, could be that the patients in the secondary infection group could have been previously infected by the same serotype (homotypic infection). Recent studies have reported the evidence of homotypic dengue re-infection [31, 32]. The findings for these studies demonstrate that, in some cases, serotype-specific immunity to DENV may be short-lived. Such cases challenge the paradigm of lifelong, serotype-specific DENV immunity following a natural infection. A greater risk to develop severe dengue is caused by heterotypic secondary DENV infection with a dengue serotype distinct from the primary infecting type [33]. Having said that, prior to the onset of the recent outbreak, DENV 1 were equally circulating with other serotypes (DENV 2 and DENV 3) from the year 2010–2012. It was only in the year 2013, there was an upsurge of DENV 2. Since the information is not available on the serotype that previously infected the patients in the secondary infection group, therefore our findings raised the possibility that these patients had been previously infected by DENV 1 also. Hence, increased disease severity was not observed. The theory of peak enhancement titer provides a second possible explanation. The enhancement of severe dengue is dependent on the pre-existing antibody titer, resulting from a different serotype infection. The pre-existing dengue antibody titer of 1: 21 to 1:80 induced high risk to develop severe dengue, whereas, a titer above 1: 320 triggered a protective effect [33]. It is more likely that the pre-existing dengue antibody titers in the secondary infection group in our study did not fall into the peak enhancement zone, thus causing less risk for severity. The IgM and IgG rapid test results were utilized to discriminate between primary and secondary infection. Several studies have evaluated the accuracy of infection status assignment using this method. One such study demonstrated a 100% sensitivity of an IgM/IgG rapid test in an attempt to distinguish between primary and secondary dengue virus infections [34]. Another study evaluated the similar rapid test kit (Dengue Duo Cassette; Panbio, Brisbane, Australia) which was also used in the present study and found that the test has good reproducibility, with the inconsistency of only 5% [35]. These findings collectively showed that the IgM/IgG rapid tests are reliable diagnostic tools for the indication of primary or secondary dengue infection.

Several strengths are inherent in this study. To the best of the researcher’s knowledge, this is the first observational study to investigate the relationship between dengue serotype and genotype with patients’ clinical manifestation during the recent outbreak peri-od among Malaysians. Additionally, our study findings may warrant further research to elucidate the strong association of dengue serotype and genotypes focusing on a larger population and localities. However, the study has limitations as well. Firstly, all retrospective studies depend on the completeness of medical data, and missing data is therefore unavoidable. In the present study, a number of subjects had to be excluded from the original count due to incompleteness of clinical data, thus resulted in a smaller sample size. Secondly, in Malaysia, many dengue specimens from hospitals were sent to the national reference laboratories for molecular diagnosis and surveillance purpose. Therefore, our collaborators from the hospitals were only able to provide those specimens that were not required for national surveillance. We need to acknowledge that there is a possibility of selection bias but, the findings in our study of resurgent DENV 1 infection during the recent outbreak period is consistent with the national surveillance of the dengue infection. In view of this, the selection bias was not substantial. Not only that, there was underrepresentation of certain serotypes in this study due to the small numbers such as DENV 4 causing it to be omitted from the statistical analysis. Lastly, the proportion of dengue subjects obtained in the year 2014 was low despite a spike in the dengue cases because the sample collection was initiated towards the end of the particular year.

Our study findings demonstrated that symptoms of dengue infected patients in Malaysia were indeed serotype and genotype-specific. DENV 1 was found to cause dengue without warning signs and mild symptoms. DENV 2 patients were more likely to present with severe dengue as compared to other serotypes. In addition, DENV 3-infected patients frequently had musculoskeletal symptoms. These findings suggested that different dengue serotypes targeted different receptors or organs to establish infection. Following our findings and to benefit from further research, we recommend continuous monitoring of dengue clinical manifestations in relation to serotype and genotype and investigate the link between DENV 3 genotype III and disease severity with larger sample size. We also propose to look into the recent prevalence of primary and secondary dengue infection among Malaysian population as this has an implication on dengue vaccine.

## Supporting information

S1 Checklist STROBE checklist. (DOCX)

S1 Table Distribution of primary and secondary infection by age group. (DOCX)

S1 File GenBank accession numbers and sequence ID. (DOC)

## References

[1] Skae FMT. Dengue fever in Penang. The British Med J. 1902;2:1581–82. 10.1136/bmj.2.2185.1581-a PMC2402112 20760524

[2] Mudin R. Dengue incidence and the prevention and control program in Malaysia. Int. Med. J. M’sia. 2015;14(1):5–9.

[3] Malaysian Remote Sensing Agency (ARSM), Ministry of Science Technology and Innovation (MOSTI) & Disease Control Division, Ministry of Health Malaysia. i-Dengue untuk komuniti. 2014. http://idengue.remotesensing.gov.my/idengue/index.php

[4] Recker M, Blyuss KB, Simmons CP, Hien TT, Wills B, Farrar J, et al Immunological serotype interactions and their effect on the epidemiological pattern of dengue. Proc R Soc B Biol Sci. 2009;276(1667):2541–8. 10.1098/rspb.2009.0331 PMC2684681 19369266

[5] Sanjeev KT, Prashant G, Vineeta K, Animesh C, Rashmi K, Mohammed YK et al Emergence of new lineage of Dengue virus 3 (genotype III) in Lucknow, India. Iranian J Microb. 2013;5(1):68–75. PMC3577557 23466682

[6] Hasitha AT, Eng EO, Duane JG, Ying Tan, Barathy l, Wahala MPB. New Dengue virus type 1 genotype in Colombo, Sri Lanka. Emerging Infec Dis. 2011;17(11):2053–55. 22099096 10.3201/eid1711.101893 PMC3310553

[7] Halsey ES, Marks MA, Gotuzzo E, Fiestas V, Suarez L, Vargas J, et al Correlation of Serotype-Specific Dengue Virus Infection with Clinical Manifestations. PLoS Negl Trop Dis. 2012;6(5):e1638 10.1371/journal.pntd.0001638 22563516 PMC3341333

[8] Thomas L, Verlaeten O, Cabie A, Kaidomar S, Moravie V, Martial J, et al Influence of the dengue serotype, previous dengue infection, and plasma viral load on clinical presentation and outcome during a dengue-2 and dengue-4 co-epidemic. Am J of Trop Med Hyg. 2008;78(6):990–98. 18541782

[9] Yung CF, Lee KS, Thein TL, Tan LK, Gan VC, Wong JGX, et al Dengue Serotype-Specific Differences in Clinical Manifestation, Laboratory Parameters and Risk of Severe Disease in Adults, Singapore. Am J of Trop Med Hyg. 2015; 92(5): 999–05. 25825386 10.4269/ajtmh.14-0628 PMC4426593

[10] Johnson BW, Russell BJ, Lanciotti RS. Serotype-Specific Detection of Dengue Viruses in a Fourplex Real-Time Reverse Transcriptase PCR Assay. J. Clin Microbiol. 2005;43(10):4977–83. 10.1128/JCM.43.10.4977-4983.2005 16207951 PMC1248506

[11] Shu PY, Su CL, Liao TL, Yang CF, Chang SF, Lin CC, et al Molecular characterization of dengue viruses imported into Taiwan during 2003–2007: geo- graphic distribution and genotype shift. Am J Trop Med Hyg. 2005;80:1039–46. 19478273

[12] Abubakar S & Shafee N. Outlook of dengue in Malaysia: a century later. Malays. J. Pathol. 2002;24:23–27. 16329552

[13] Mohd-Zaki AH, Brett J, Ismail E, L’Azou M. Epidemiology of Dengue Disease in Malaysia (2000–2012): A Systematic Literature Review. PLoS Negl Trop Dis. 2014; 8(11):e3159 10.1371/journal.pntd.0003159 25375211 PMC4222702

[14] Chew MH, Rahman MM, Jekip J, Hassan MR, Isahak I. All serotypes of dengue viruses circulating in Kuala Lumpur, Malaysia. Current Research Journal of Biological Sciences. 2012;4(2):229–34.

[15] Tan KK, Zulkifle NI, Abd-Jamil J, Sulaiman S, Yaacob CN, Azizan NS, et al Disruption of predicted dengue virus type 3 major outbreak cycle coincided with switching of the dominant circulating virus genotype. Infect Genet Evol. 2017;54:271–75. 10.1016/j.meegid.2017.07.008 28698156

[16] Dash PK, Sharma S, Srivastava A, Santhosh SR, Parida MM, Neeraja M, et al Emergence of dengue virus type 4 (genotype I) in India. Epidemiology and Infection. 2011;139(6):857–61. 10.1017/S0950268810001706 20670467

[17] Sun J, Wu D, Zhou H, Zhang H, Guan D, He X, et al The epidemiological characteristics and genetic diversity of dengue virus during the third largest historical outbreak of dengue in Guangdong, China, in 2014. J Infect. 2016;72(1):80–90. 10.1016/j.jinf.2015.10.007 26546854

[18] Teoh BT, Sam SS, Tan KK, Johari J, Shu M-H, Danlami MB, et al Dengue virus type 1 clade replacement in recurring homotypic outbreaks. BMC Evol Biol. 2013;13:213 10.1186/1471-2148-13-213 24073945 PMC3850903

[19] Nurulfateha M, Shaharom NAC, Norlin AL, Nur Nadiah Y. Serotype and phylogenetic analysis of dengue virus–Johor 2012–2014. Report from Johor Bahru Public Health Laboratory. http://jknj.moh.gov.my/jsm

[20] Kumaria R. Correlation of disease spectrum among four dengue serotypes: a five years hospital based study from India. Braz J Infect Dis. 2010;14(2):141–46. 20563439

[21] Oliveira MF, Galvao Araujo JM, Ferreira OC Jr, Ferreira DF, Lima DB, Santos FB, et al Two lineages of dengue virus type 2, Brazil: Emerg Infect Dis. 2010;16(3):576–78. 10.3201/eid1603.090996 20202456 PMC3322019

[22] Vaughn DW, Green S, Kalayanarooj S, Innis BL, Nimmannitya S, Suntayakorn S, et al Dengue viremia titer, anitibody response pattern and virus serotype correlate with disease severity. The Journal of Infectious Diseases. 2000;181:2–9. 10.1086/315215 10608744

[23] Soo KM, Khalid B, Ching SM, Chee HY. Meta-analysis of dengue severity during infection by different dengue virus serotypes in primary and secondary infections. PLoS One. 2016;11(5):4–14. 10.1371/journal.pone.0154760 PMC4877104 27213782

[24] Kalayanarooj S, Nimmannitya S. Clinical and laboratory presentations of dengue patients with different serotypes. WHO Dengue Bulletin. 2000; 24:53–59.

[25] Martins VDCA, De Bastos MS, Ramasawmy R, De Figueiredo RP, Gimaque JBL, Braga WSM, et al Clinical and virological descriptive study in the 2011 outbreak of dengue in the Amazonas, Brazil. PLoS One. 2014;9(6):e100535vn 24978469 10.1371/journal.pone.0100535 PMC4076277

[26] Chan KS, Chang JS, Chang K, Lin CC, Huang JH, Lin WR, et al Effect of serotypes on clinical manifestations of dengue fever in adults. J Microbiol Immunol Infect. 2009;42:471–78. 20422131

[27] Chew CH, Woon YL, Amin F, Adnan TH, Abdul Wahab AH, Ahmad ZE, et al Rural-urban comparisons of dengue seroprevalence in Malaysia. BMC Public Health. 2016;16(1):1–9. 27538986 10.1186/s12889-016-3496-9 PMC4991088

[28] Jamaiah I, Rohela M, Nissapatorn V, Hiew FT, Mohammad Halizam A, Noor Liani H, et al Retrospective study of dengue fever (DF) and dengue hemorrhagic fever (DHF) patients at University Malaya Medical Center, Kuala Lumpur, Malaysia in the year 2005. Southeast Asian J Trop Med Public Health. 2007; 38(1).

[29] Tee HP, How SH, Jamalludin AR, Safhan MN, Sapian MM, Kuan YC, et al Risk factors associated with development of dengue haemorrhagic fever or dengue shock syndrome in adults in Hospital Tengku Ampuan Afzan Kuantan. Med J Malaysia. 2009; 64(4):316–20. 20954558

[30] Anantapreecha S, Chanama S, A-Nuegoonpipat A, Naemkhunthot S, Sa-Ngasang A, Sawanpanyalert P, et al Serological and virological features of dengue fever and dengue haemorrhagic fever in Thailand from 1999 to 2002. Epidemiol Infect. 2005;133:503–07. 15962557 10.1017/s0950268804003541 PMC2870274

[31] Waggoner JJ, Balmaseda A, Gresh L, Sahoo MK, Montoya M, Wang C, et al Homotypic Dengue Virus Reinfections in Nicaraguan Children. J Infect Dis. 2016;214(7):986–93. 10.1093/infdis/jiw099 26984144 PMC5021223

[32] Buddhari D, Aldstadt J, Endy TP, Srikiatkhachorn A, Thaisomboonsuk B, Klungthong C, et al Dengue Virus Neutralizing Antibody Levels Associated with Protection from Infection in Thai Cluster Studies. PLoS Negl Trop Dis. 2014;8(10). 10.1371/journal.pntd.0003230 PMC4199527 25329173

[33] Katzelnick LC, Gresh L, Halloran ME, Mercado JC, Kuan G, Gordon A, et al Antibody-dependent enhancement of severe dengue disease in humans. 2017;932:929–32. 10.1126/science.aan6836 PMC5858873 29097492

[34] Vaughn DW, Nisalak A, Kalayanarooj S, Solomon T, Dung NM, Cuzzubbo A, et al Evaluation of a rapid immunochromatographic test for diagnosis of dengue virus infection. J Clin Microbiol. 1998;36(1):234–8. 9431954 10.1128/jcm.36.1.234-238.1998 PMC124841

[35] Lima JRC, Rouquayrol MZ, Callado MRM, Guedes MIF, Pessoa C. Interpretation of the presence of IgM and IgG antibodies in a rapid test for dengue: analysis of dengue antibody prevalence in Fortaleza City in the 20th year of the epidemic. Rev Soc Bras Med Trop. 2012;45(2):163–7. 22534985 10.1590/s0037-86822012000200005

