# Supplementary material for: Clinical manifestations of dengue in relation to dengue serotype and genotype in Malaysia: A retrospective observational study
Source: PLoS Negl Trop Dis. 2018 Sep 18;12(9):e0006817. doi: 10.1371/journal.pntd.0006817 (PMC6161924; doi:10.1371/journal.pntd.0006817)
Supplement: S1 Table — (DOCX) [file pntd.0006817.s002.docx]

| Age groups  (years) | Primary infection (n=97)  % (n) | Secondary infection (n=4) % (n) |
| --- | --- | --- |
| ≤20 (n=52) | 100 (52/52) | 0 (0/52) |
| 21-40 (n=38) | 94.7 (36/38) | 5.3 (2/38) |
| 41-60 (n=8) | 75.0 (6/8) | 28.6 (2/8) |
| ≥61 (n=3) | 100 (3/3) | 0 (0/3) |
