# Supplementary material for: Clinical manifestations of dengue in relation to dengue serotype and genotype in Malaysia: A retrospective observational study
Source: PLoS Negl Trop Dis. 2018 Sep 18;12(9):e0006817. doi: 10.1371/journal.pntd.0006817 (PMC6161924; doi:10.1371/journal.pntd.0006817)
Supplement: S1 File — (DOC) [file pntd.0006817.s003.doc]

**S1 File**

The full details of the sequences are as follows: GenBank Accession Numbers and Sequence ID:

MG450795:D1/Malaysia/PUR/765027/15; MG450796:D1/Malaysia/5348/16; MG450797:D1/Malaysia/5731/16; MG450798:D1/Malaysia/5473/16; MG450799:D1/Malaysia/SMN/734726/15;MG450800:D1/Malaysia/5474/16; MG450801:D1/Malaysia/6489/16; MG450802:D1/Malaysia/HCX/754370/15; MG450803:D1/Malaysia/SUL/774863/16; MG450804:D1/Malaysia/WM/384125/15; MG450805:D1/Malaysia/AIM/735889/15; MG450806:D1/Malaysia/FTN/395574/15; MG450807:D1/Malaysia/LMS/771766/16; MG450808:D1/Malaysia/BL/739026/15; MG450809:D1/Malaysia/ROB/735987/15; MG450810:D1/Malaysia/SOLH/764795/15; MG450811:D1/Malaysia/SNG/734775/15; MG450812:D1/Malaysia/ORL/755685/15; MG450813:D1/Malaysia/SROM/754816/15; MG450814:D1/Malaysia/KOK/730226/15; MG450815:D1/Malaysia/SJUN/743885/16; MG450816:D1/Malaysia/LYK/670912/14; MG450817:D1/Malaysia/NSYZ/460183/16; MG450818:D1/Malaysia/BOS/211594/16; MG450819:D1/Malaysia/AKF/507767/16; MG450820:D1/Malaysia/IVN/640495/15; MG450821:D1/Malaysia/KRS/6888553/15; MG450822:D1/Malaysia/SYM/439947/15;

MG450823:D1/Malaysia/ALF/761464/15; MG450824:D1/Malaysia/ALM/584480/14; MG450825:D1/Malaysia/SHAF/684800/15; MG450826:D1/Malaysia/FZ/652208/15; MG450827:D1/Malaysia/CHW/737983/15; MG450828:D1/Malaysia/SYR/73636/15; MG450829:D1/Malaysia/BAT/695309/15; MG450830:D1/Malaysia/MUS/157847/14; MG450831:D1/Malaysia/SND/682748/14; MG450832:D1/Malaysia/SYFI/688771/15; MG450833:D1/Malaysia/TNG/690232/15; MG450834:D1/Malaysia/HAI/476062/16; MG450835:D1/Malaysia/DAN/254612/16; MG450836:D1/Malaysia/LYA/691712/15; MG450837:D1/Malaysia/ZAF/176903/15; MG450838:D1/Malaysia/ISN/701164/15; MG450839:D1/Malaysia/LAT/683800/14; MG450840:D1/Malaysia/HAM/695815/15; MG450841:D1/Malaysia/WNG/768882/16; MG450842:D1/Malaysia/ZM/633513/15; MG450843:D1/Malaysia/CHOI/697657/15; MG450844:D1/Malaysia/MRY/402550/15; MG450845:D1/Malaysia/AD/696600/15; MG450846:D1/Malaysia/YER/759658/15; MG450847:D1/Malaysia/561064/17; MG450848:D1/Malaysia/172081/17; MG450849:D1/Malaysia/5793/17; MG450850:D1/Malaysia/WTO/734611/15; MG450851:D1/Malaysia/5878/17; MG450852:D1/Malaysia/5054/17; MG450853:D1/Malaysia/5875/16; MG450854:D1/Malaysia/YZD/393714/15; MG450855:D1/Malaysia/5696/16;MG450856:D1/Malaysia/ ASDK/698207/15; MG450857:D1/Malaysia /HLZ/742536/15; MG450858:D1/Malaysia/MOS/776274/16; MG450859:D2/Malaysia/5129/16; MG450860:D2/Malaysia/AMR/747585/15; MG450861:D2/Malaysia/SN/688163/15; MG450862:D2/Malaysia/KZ/742808/15; MG450863:D2/Malaysia/5222/16; MG450864:D2/Malaysia/5069/16; MG450865:D2/Malaysia/5276/16;MG450866:D2/Malaysia/5995/16;

MG450867:D2/Malaysia/5718/16; MG450868:D2/Malaysia/INM/247146/15; MG450869:D2/Malaysia/AIM/768397/15; MG450870:D2/Malaysia/HOM/733682/15; MG450871:D2/Malaysia/BAG/680429/14; MG450872:D2/Malaysia/SUG/92678/15; MG450873:D2/Malaysia/SHIN/737431/15; MG450874:D2/Malaysia/WCK/738138/15; MG450875:D2/Malaysia/5373/16; MG450876:D2/Malaysia/5182/16; MG450877:D2/Malaysia/SAL/248126/15; MG450878:D2/Malaysia/ZUL/746682/15; MG450879:D2/Malaysia/PMY/753568/15; MG450880:D2/Malaysia/HAF/676583/14; MG450881:D2/Malaysia/870855/17; MG450882:D2/Malaysia/557599/17; MG450883:D2/Malaysia/177613/17; MG450884:D2/Malaysia/5874/17; MG450885:D2/Malaysia/558764/17; MG450886:D2/Malaysia/550620/17; MG450887:D2/Malaysia/KKM/683104/14; MG450888:D2/Malaysia/CHT/746049/15; MG450889:D2/Malaysia/DOR/480537/15; MG450890:D2/Malaysia/IRW/243588/16; MG450891:D3/Malaysia/LTN/747609/15; MG450892:D3/Malaysia/PLN/224544/15; MG450893:D3/Malaysia/5117/16; MG450894:D3/Malaysia/5169/16; MG450895:D3/Malaysia/5303/16; MG450896:D3/Malaysia/GHZ/685569/14; MG450897:D3/Malaysia/BND/674434/14; MG450898:D3/Malaysia/6069/16; MG450899:D3/Malaysia/SUJ/685338/14; MG450900:D3/Malaysia/SYHIS/688771/15;

MG450901:D3/Malaysia/ANS/682233/14; MG450902:D3/Malaysia/STO/697902/15; MG450903:D3/Malaysia/RIZ/694082/15; MG450904:D3/Malaysia/FIR/579795/15; MG450905:D3/Malaysia/AMI/680448/14; MG450906:D3/Malaysia/AIA/729808/15; MG450907:D3/Malaysia/5735/17; MG450908:D3/Malaysia/5185/17; MG450909:D3/Malaysia/6292/17; MG450910:D3/Malaysia/5464/17; MG450911:D4/Malaysia/5657/16; MG450912:D4/Malaysia/CKM/676500/14;

MG450913:D4/Malaysia/DG431/14; MG450914:D4/Malaysia/DG373/14

MG450913:D4/Malaysia/DG431/14; MG450914:D4/Malaysia/DG373/14.
